# Structural and Antihypertensive Properties of Enzymatic Hemp Seed Protein Hydrolysates

**DOI:** 10.3390/nu7095358

**Published:** 2015-09-10

**Authors:** Sunday A. Malomo, John O. Onuh, Abraham T. Girgih, Rotimi E. Aluko

**Affiliations:** 1 Department of Human Nutritional Sciences, University of Manitoba, Winnipeg, MB R3T 2N2, Canada; E-Mails: malomos@myumanitoba.ca (S.A.M.); onuhj@myumanitoba.ca (J.O.O.); umgirgia@myumanitoba.ca (A.T.G.); 2 The Richardson Centre for Functional Foods and Nutraceuticals, University of Manitoba, Winnipeg, MB R3T 2N2, Canada

**Keywords:** hemp seed, protein hydrolysate, renin, angiotensin converting enzyme, degree of hydrolysis, fluorescence intensity, systolic blood pressure, spontaneously hypertensive rats

## Abstract

The aim of this work was to produce antihypertensive protein hydrolysates through different forms of enzymatic hydrolysis (2% pepsin, 4% pepsin, 1% alcalase, 2% alcalase, 2% papain, and 2% pepsin + pancreatin) of hemp seed proteins (HSP). The hemp seed protein hydrolysates (HPHs) were tested for *in vitro* inhibitions of renin and angiotensin-converting enzyme (ACE), two of the enzymes that regulate human blood pressure. The HPHs were then administered orally (200 mg/kg body weight) to spontaneously hypertensive rats and systolic blood pressure (SBP)-lowering effects measured over a 24 h period. Size exclusion chromatography mainly showed a 300–9560 Da peptide size range for the HPHs, while amino acid composition data had the 2% pepsin HPH with the highest cysteine content. Fluorescence spectroscopy revealed higher fluorescence intensities for the peptides when compared to the unhydrolyzed hemp seed protein. Overall, the 1% alcalase HPH was the most effective (*p* < 0.05) SBP-reducing agent (−32.5 ± 0.7 mmHg after 4 h), while the pepsin HPHs produced longer-lasting effects (−23.0 ± 1.4 mmHg after 24 h). We conclude that an optimized combination of the fast-acting HPH (1% alcalase) with the longer-lasting HPHs (2% and 4% pepsin) could provide daily effective SBP reductions.

## 1. Introduction

An increased rate of high blood pressure (BP) has led to critical hypertensive conditions in most nations and is responsible for ~45%–51% of total global deaths [1]. BP is said to be under normal and adequate control when the systolic and diastolic values are 140 and 90 mm Hg, respectively [1]. Occurrence of elevated BP is a known risk factor for the development of several cardiovascular diseases like coronary heart disease, heart failure, stroke, peripheral arterial disease, and renal failure [2]. Antihypertensive drug therapy (a key aspect of hypertension management), which is currently being employed to screen, treat and control high BP in order to reduce the incidence of hypertension, has created a significant healthcare burden for several nations [3]. For instance, in order to control this public health burden, the USA incurred a direct and indirect cost of about US$93.5 billion in 2010 for hypertension awareness, treatment and control [3]. Likewise, increasingly significant health, policy and financing challenges have been recorded in some countries, which inevitably create unsustainable pharmaceutical costs for government [4].

The renin–angiotensin aldosterone system (RAAS) is the primary physiological pathway that has been described for BP regulation and management [5]. To control BP, renin is synthesized in the kidneys and then released into the blood circulatory system where it cleaves the N-terminal region of angiotensinogen to produce a decapeptide, angiotensin (AT)-I [6]. AT-I further circulates in the blood until its *C*-terminal dipeptide residue is cleaved by angiotensin I-converting enzyme (ACE) to form an octapeptide AT-II (a potent vasoconstrictor). The pharmaceutical industry has historically exploited ACE inhibition to produce commercial antihypertensive drugs like captopril, enalapril, and lisinopril [7]. Meanwhile, a comparative reduction in elevated BP that could lead to hypertension treatment other than ACE inhibition has been suggested for research [8]. Such alternative antihypertensive compounds target renin, and bioactive peptides from plant proteins have been shown to have renin- and ACE-inhibitory properties [9,10,11,12,13,14,15]. The need to reduce negative side effects (nausea, vomiting, dry cough) of antihypertensive therapy has spurred research into alternative natural sources of effective compounds such as food protein-derived peptides. Thus there are various reports that have shown bioactive peptides to be potential antihypertensive agents [9,10,11,12,13,14,16,17,18,19,20,21,22,23,24].

Hemp seed protein (HSP), which is obtained from the industrial production of edible oil, has been shown to be a suitable raw material for antihypertensive peptide production [12,14]. However, previous works have focused solely on the use of simulated gastrointestinal digestion as a tool to produce blood-pressure-reducing peptides from hemp seed proteins. Therefore, there is a need to expand the scope of hemp seed antihypertensive research by examining the potential use of other commercially available enzymes as suitable proteolytic agents. This is because past studies have demonstrated the possibility of generating antihypertensive peptides with varying potencies simply by using different enzymes and enzyme:substrate ratios [9,17,22,25,26]. This work aimed to determine the structural properties and BP-lowering effects of different hemp seed protein hydrolysates (HPHs) produced using four enzymes and different enzyme:substrate ratios. Specifically, the HPHs were tested for *in vitro* inhibitions of renin and ACE activities, which were then related to observed BP-lowering effects after oral administration to spontaneously hypertensive rats.

## 2. Experimental Section

### 2.1. Hemp Seed Products and Chemical Reagents

Hemp seed protein meal (HPM, 37% protein content) was a gift from Hemp Oil Canada (St. Agathe, MB, Canada). Renin was purchased from Cayman Chemical Co. (Ann Arbor, MI, USA) while other enzymes such as pepsin, pancreatin, papain, alcalase, and ACE (rabbit lung) were purchased from Sigma-Aldrich (St. Louis, MO, USA). Other analytical-grade reagents were obtained from Fisher Scientific (Oakville, ON, Canada).

### 2.2. Preparation of Hemp Seed Protein Isolates (HPI)

HPI was produced from HPM according to the method previously described [12] with slight modifications. HPM was dispersed in a glass beaker that contained deionized water (1:20, *w*/*v*) and the dispersion was adjusted to pH 8.0 using 2 M NaOH to solubilize the proteins while stirring at 37 °C for 2 h; this was followed by centrifugation (7000× *g*, 60 min at 4 °C). The first precipitate was discarded and the supernatant filtered with cheese-cloth, adjusted to pH 5.0 with 2 M HCl to insolubilize the proteins and then centrifuged (7000× *g*, 60 min at 4 °C). The second precipitate was re-dispersed in deionized water, adjusted to pH 7.0 with 2 M NaOH and freeze-dried to obtain the HPI. The protein concentration of the HPI was determined using the modified Lowry method [27].

### 2.3. Preparation of Enzymatic Hemp Seed Protein Hydrolysates (HPHs)

Hydrolysis of the HPI was conducted using each of the following enzyme and reaction conditions as previously reported [9] with slight modifications: alcalase (50 °C, pH 8.0, 4 h); papain (65 °C, pH 6, 4 h); and PP (pepsin at 37 °C, pH 2.0, 2 h followed by pancreatin at 37 °C, pH 7.5, 4 h). HPI (5%, *w*/*v*, protein weight basis) was suspended in deionized water in a glass beaker equipped with a stirrer, heated to the appropriate temperature and adjusted to the appropriate pH value prior to addition of the proteolytic enzymes. Proteases were added to the HPI slurry using optimized enzyme-to-substrate ratios (E/S), *viz* 1:100 (alcalase), 2:100 (pepsin, alcalase, papain, PP), and 4:100 (pepsin), based on HPI protein content. During digestion, the required pH was kept constant by addition of NaOH, after which the enzymes were inactivated by adjusting to pH 4.0 with 2 M HCl followed by immersing the reaction vessel in boiling water bath for 10 min. The undigested proteins were precipitated by centrifugation (8000× *g*, 60 min at 4 °C) after cooling and the supernatant (contains target peptides) was freeze-dried as the HPH, which was then stored at −20 °C until needed for further analysis. The protein contents of the freeze-dried HPH was determined using the modified Lowry method [27].

### 2.4. Determination of Degree of Hydrolysis

The percent degree of hydrolysis (DH) of HPHs was determined according to the trinitrobenzene sulfonic acid method as previously described [28]. Briefly, HPI was digested under vacuum with 6 M HCl for 24 h and the digest was used to determine total amino groups as l-leucine equivalent. The DH was calculated as the percentage ratio of the leucine equivalent of HPHs to that of HPI.

### 2.5. Amino Acid Composition Analysis

The amino acid profiles of HPI and HPHs were determined using the HPLC S4300 Amino Acid Analyzer, (Sykam Mfd Co., Eresing, Bavaria, Germany) according to the method previously described [29] after samples were digested with 6 M HCl for 24 h. The cysteine and methionine contents were determined after performic acid oxidation [30] and the tryptophan content was determined after alkaline hydrolysis [31].

### 2.6. Analysis of Molecular Weight Distribution

Molecular weight (MW) distribution of HPH peptides was determined by size exclusion chromatography (SEC) as previously described [9] with slight modifications, using an AKTA FPLC system (GE Healthcare, Montreal, PQ, Canada) equipped with a Superdex Peptide 10/300 GL column (10 × 300 mm), and UV detector (λ = 214 nm). An aliquot (100 µl) of the sample (5 mg/mL in 50 mM phosphate buffer, pH 7.0 containing 0.15 M NaCl) was loaded onto the column and elution was performed at room temperature using the phosphate buffer at a flow rate of 0.5 mL/min. The column was calibrated with cytochrome C (12,384 Da), Aprotinin (6512 Da), vitamin B_12_ (1855 Da), and Glycine (75 Da) as the standard proteins. Peptide sizes of the samples were estimated from a plot of log MW *versus* elution volume of the standard proteins.

### 2.7. Intrinsic Fluorescence

Fluorescence intensity spectra of protein and peptide samples were obtained using a previously described method [32] on a Jasco FP-6300 spectrofluorimeter (Jasco, Tokyo, Japan) at 25 °C in a 1-cm path length cuvette. The sample stock solution was prepared as 10 mg/mL in 0.1 M sodium phosphate buffer, pH 7.0; this was followed by centrifugation and determination of protein content of the supernatant. After diluting the supernatant to 0.002% protein content (*w*/*v*), the fluorescence spectra was recorded at 280 nm excitation wavelengths and 300 to 500 nm emission. The final fluorescence emission spectrums of each sample were obtained after subtraction of the buffer emission spectrum.

### 2.8. ACE Inhibition Assay

The ability of HPHs to inhibit *in vitro* ACE activity was measured according to a spectrophotometric method using synthetic *N*-[3-(2-Furyl)acryloyl]-l-phenylalanyl-glycyl-glycine (FAPGG) as the substrate (Sigma-Aldrich, St. Louis, MO, USA) [33]. Briefly, 1 mL of 0.5 mM FAPGG (dissolved in 50 mM Tris-HCl buffer containing 0.3 M NaCl, pH 7.5) was mixed with 20 µL of ACE (20 mU final reaction activity) and 200 µL sample dissolved in same buffer. The rate of decrease in absorbance at 345 nm was recorded for 2 min at room temperature using Varian Cary 50-UV/Visible spectrophotometer (Varian Inc., Belrose, NSW, Australia). The buffer was used instead of sample solutions in the blank experiment. The concentration of sample that inhibited ACE activity by 50% (IC_50_) was calculated from a non-linear regression plot of percentage ACE activity *versus* sample concentrations. ACE activity was expressed as the rate of reaction (ΔA/min), and inhibitory activity was calculated as:

(1) ACE inhibition (%) = 1 – (ΔA·min^−1^_(sample)_/ ΔA·min^−1^_(blank)_) × 100

where ΔA·min^−1^_(sample)_ and ΔA·min^−1^_(blank)_ represent ACE activity in the presence and absence of the HPHs, respectively.

### 2.9. Renin Inhibition Assay

*In vitro* inhibition of human recombinant renin activity by HPHs was conducted using the Renin Inhibitor Screening Assay Kit (Cayman Chemical Co., Ann Arbor, MI, USA) according to the method previously described [9]. Prior to the assay, renin buffer was diluted in 50 mM Tris–HCl, pH 8.0, containing 100 mM NaCl. The renin protein solution was diluted 20 times with the assay buffer before use and pre-warmed to 37 °C prior to initiating the reaction in a fluorometric microplate reader (Spectra MAX Gemini, Molecular Devices, Sunnyvale, CA, USA) maintained at 37 °C. Before the reaction, (i) 20 µL substrate, 160 µL assay buffer, and 10 µL Milli-Q water were added to the background wells; (ii) 20 µL substrate, 150 µL assay buffer, and 10 µL Milli-Q water were added to the blank wells; and (iii) 20 µL substrate, 150 µL assay buffer, and 10 µL sample were added to the inhibitor wells. The reaction was initiated by adding 10 µL renin to the blank and sample wells. The microplate was shaken for 10 sec to mix and incubated at 37 °C for 15 min, and the fluorescence intensity (FI) was recorded using excitation and emission wavelengths of 340 and 490 nm, respectively. The concentration of sample that inhibited renin activity by 50% (IC_50_) was calculated from a non-linear regression plot of percentage renin activity *versus* peptide concentrations. The percentage renin inhibition was calculated as follows:

(2) $$\text{Renin inhibition }\left(\text{\%}\right)=\frac{{\text{FI}}_{\left(\text{blank}\right)}-{\text{FI}}_{\left(\text{sample}\right)}}{{\text{FI}}_{\left(\text{blank}\right)}}\times 100$$

### 2.10. BP-lowering Effect of Peptides in Spontaneously Hypertensive Rats (SHRs)

Animal experiments were carried out following the Canadian Council on Animal Care Ethics guidelines with a protocol approved by the University of Manitoba Animal Protocol and Management Review Committee. The 30-week old male SHRs (Charles River Laboratories, Montreal, QC, Canada) with 340–380 g body weight (bw) were kept in the Animal Housing Facility at the Richardson Centre for Functional Foods and Nutraceuticals, under a 12 h day and night cycle at 22 ± 2 °C and were fed regular diet and tap water. The rats were divided into three groups with 4 rats per group: HPH (test sample), captopril (positive control) and phosphate buffered saline (PBS, pH 7.4) as the negative control. HPHs (each at 200 mg/kg bw) and captopril (10 mg/kg bw) were dissolved in 1 mL PBS and administered to the SHRs by oral gavage followed by measurement of systolic blood pressure (SBP) at 2, 4, 6, 8, and 24 h using the tail-cuff method in slightly anesthetized rats as previously described [12]. Prior to sample administration, the baseline (time-zero) SBP was determined. The change in SBP (ΔSBP, mmHg) was determined by subtracting the baseline data from the data obtained at different time points. Oral gavage was used to ensure all rats received the same amount of the HPHs while the 200 mg/kg rat bw translates to 32.43 mg/kg bw (~2 g/day for 60 kg) in an adult human being [34].

### 2.11. Statistical Analysis

Triplicate replications were used to obtain mean values and standard deviations. Statistical analysis was performed with SAS (Statistical Analysis Software 9.2, SAS Institute Inc, Cary, NC, USA). using one-way ANOVA. Duncan’s multiple-range test was carried out to compare the mean values for samples with significant differences taken at *p* < 0.05.

## 3. Results

### 3.1. Amino Acid Composition of HPI and HPHs

Table 1 shows that the protein isolate and hydrolyzed samples had very similar amino acid composition, which is consistent with the hydrolyzed samples being derived from same protein starting material. However, the 2% pepsin hydrolysate had very low cysteine content (0.12%) in comparison to the 1.2%–1.43% for the other samples. The 2% papain HPH had the highest proline content though differences were not significant (*p* > 0.05).

### 3.2. DH and Size Exclusion Chromatography Analysis of HPHs

**Figure 1:**
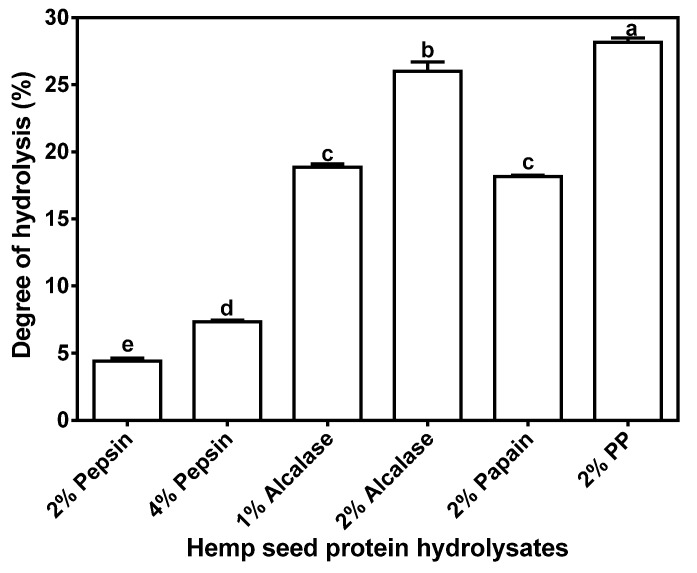
Degree of hydrolysis of different enzymatic hemp protein hydrolysates.

**Figure 2:**
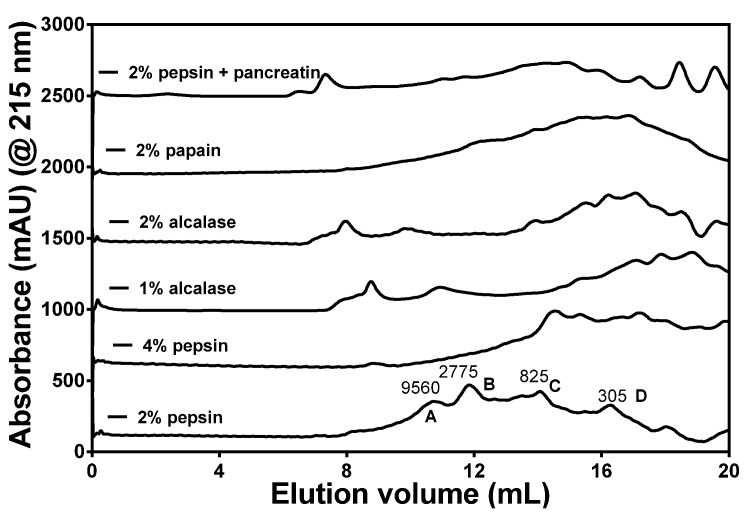
Gel-permeation chromatograms of different enzymatic hemp seed protein hydrolysates at different concentrations after passage through a Superdex Peptide 10/300 GL column.

Extent of hydrolysis can be estimated from the DH values, which can be used an indication of peptide chain length; higher and lower values indicate mean shorter and longer lengths, respectively. As expected, the PP digest had significantly (*p* < 0.05) higher DH (28.16% ± 0.34%) than the other enzyme digests (Figure 1), probably due to the exo- and endo-proteinase activities of the enzyme preparations. Similar to our results, a flavourzyme protein hydrolysate was shown to exhibit the highest DH, which was attributed to the presence of endo- and exo- proteinase activities in this enzyme [35]. The peptide sizes were estimated by SEC and the results showed distribution into four major peaks with approx. MW ranging from 300 to 9560 Da (Figure 2). These peptide peaks were most noticeable for the 2% pepsin HPH; increasing the enzyme concentration to 4% led to reduced intensities of the bigger peptide peaks (2775 and 9560 Da). The alcalase and PP HPHs contained a minor peak with size >9560 Da (8–9 mL elution volume), which indicates a polypeptide that is resistant to digestion by these enzymes. Overall, the data show that the HPI was highly susceptible to proteolysis and most of the liberated peptides were <9650 Da in size.

### 3.3. Intrinsic Fluorescence Properties

**Figure 3:**
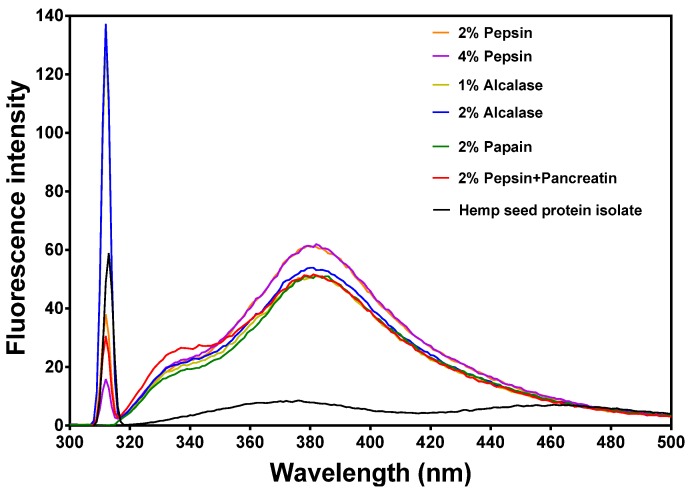
Intrinsic fluorescence properties of different enzymatic hemp protein hydrolysates.

Figure 3 shows two main peaks at 317 nm and 378 nm, which represent tyrosine and phenylalanine, respectively, in a hydrophilic environment because the observed wavelengths are longer than the normal 303 nm and 350 nm, respectively. There was also a tryptophan shoulder peak at 338 nm, which suggests some tryptophan molecules in a more hydrophobic environment but with reduced fluorescence intensity. All the protein hydrolysates had higher fluorescence intensity (FI) values than the undigested hemp seed protein isolate. The tyrosine and tryptophan peaks were each more intense for the 1% alcalase hydrolysate than the 2% alcalase hydrolysate. The 2% and 4% pepsin hydrolysates showed the most intense tryptophan 378 nm peak while the 2% alcalase had the most intense tyrosine 317 nm peak.

**Table 1:** Percentage amino acid composition of hemp seed protein isolate (HPI) and enzymatic protein hydrolysates.

Amino Acid	HPI	2% Pepsin	4% Pepsin	1% Alcalase	2% Alcalase	2% Papain	2% Pepsin + Pancreatin	Mean ± SD	*p*-Value
Asx ^a^	11.31	11.36	11.37	11.51	11.55	11.18	10.74	11.29 ± 0.27	1.00
Thr	3.42	2.92	2.85	2.89	2.78	2.93	2.82	2.95 ± 0.22	0.14
Ser	5.58	5.98	5.95	6.10	6.09	6.00	5.84	5.94 ± 0.18	1.00
Glx ^b^	19.10	19.36	19.28	19.63	19.64	19.56	19.06	19.38 ± 0.24	1.00
Pro	4.30	4.94	4.74	4.83	4.75	5.23	5.17	4.85 ± 0.31	1.00
Gly	4.31	3.82	3.84	3.73	3.70	3.63	3.56	3.80 ± 0.25	1.00
Ala	3.81	4.79	4.76	4.53	4.60	4.75	4.48	4.53 ± 0.34	0.32
Cys	1.25	0.12	1.34	1.20	1.22	1.31	1.43	1.13 ± 0.45	0.50
Val	4.66	4.60	4.33	4.25	4.20	4.36	4.71	4.45 ± 0.21	0.32
Met	1.92	2.26	2.18	2.28	2.24	2.34	2.21	2.20 ± 0.14	1.00
Ile	3.63	3.17	3.04	2.95	2.90	3.03	3.33	3.15 ± 0.26	0.14
Leu	6.46	6.64	6.48	6.38	6.39	6.35	6.60	6.48 ± 0.11	1.00
Tyr	3.61	3.49	3.39	3.39	3.38	3.28	3.62	3.45 ± 0.13	1.00
Phe	4.72	4.65	4.52	4.45	4.48	4.44	4.80	4.58 ± 0.14	0.32
His	3.15	3.09	3.13	3.12	3.13	3.02	3.00	3.10 ± 0.06	0.14
Lys	2.73	3.19	3.32	2.96	3.03	3.35	3.33	3.13 ± 0.23	0.14
Arg	15.06	14.63	14.47	14.77	14.80	14.24	14.21	14.60 ± 0.31	1.00
Trp	1.00	1.01	1.01	1.05	1.11	1.00	1.08	1.04 ± 0.04	0.50

^a^ Asx: aspartic acid + asparagine; ^b^ Glx: glutamic acid + glutamine

### 3.4. In Vitro Inhibition of ACE and Renin Activities of HPHs

**Figure 4:**
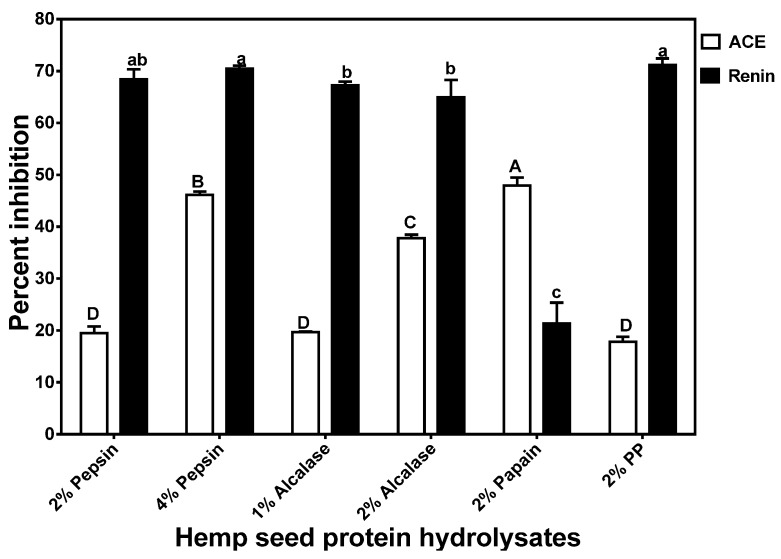
Inhibition of renin and angiotensin converting enzyme (ACE) by enzymatic hemp protein hydrolysates.

**Figure 5:**
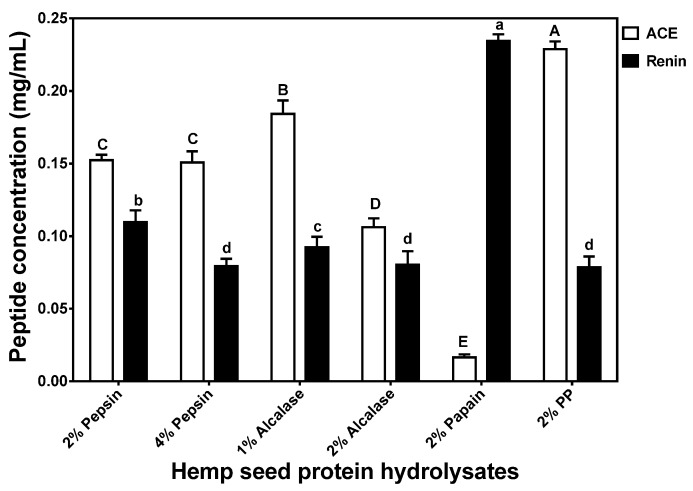
Peptide concentrations that inhibited 50% activity (IC_50_) of renin and angiotensin converting enzyme (ACE).

The 2% Papain HPH had a significantly (*p* < 0.05) higher ACE-inhibitory activity than the other HPHs when tested at 0.1 mg/mL (Figure 4). The highest ACE-inhibition value (48%) recorded in this current study is lower than the 52%–80% reported for several alcalase-treated *Parkia speciosa* seed protein hydrolysates [24] at a similar peptide concentration. However, the 48% ACE inhibition obtained for the 2% papain HPH is similar to the 50% reported for various sweet potato protein digests at 0.13–0.15 mg/mL peptide concentrations [36]. The percent ACE-inhibitory activities were directly reflected in the IC_50_ values, which were lowest (most active) for 2% papain and highest (least active) for 2% PP (Figure 5). The IC_50_ values obtained in this work (0.016 to 0.228 mg/mL) are lower and reflect higher ACE-inhibitory potency when compared to the 0.158–1.083 mg/mL single-enzyme catalyzed sweet potato protein hydrolysates [36]. The HPH IC_50_ values are also lower than previously reported values for protein hydrolysates from Adzuki bean albumin, globulin, or glutelins protein fractions [37] and canary seed [38].

Figure 4 shows that with the exception of the 2% papain, the HPHs had very high (>50%) renin-inhibitory activities and renin inhibition was higher than ACE inhibition. The noticeable higher renin-inhibitory activities in these HPHs suggest the presence of peptides that interact strongly with renin. The renin inhibition values reported in this study (with the exception of 2% papain) are higher than those (<50%) reported for macroalgae protein hydrolysates [39,40], which were performed at a higher (1 mg/mL) peptide concentration. The current results are different from our previous works that showed hemp seed [12] and canola [22] protein hydrolysates inhibit ACE activity more than renin activity. However, He *et al.* [10] showed than two HPLC fractions of rapeseed protein hydrolysate had higher renin inhibition than ACE inhibition. As shown in Figure 5, renin-inhibitory IC_50_ values also followed percent inhibitory values whereby the 2% PP, 2% alcalase and 4% pepsin HPHs had the lowest value (0.079 mg/mL) while the 2% papain had the highest (0.23 mg/mL). The renin-inhibitory IC_50_ values obtained in this work are lower than previously reported values (>0.8 mg/mL) for flaxseed [41] and hemp seed [12] protein hydrolysates.

### 3.5. In vivo Reduction of Blood Pressure by HPHs

**Figure 6:**
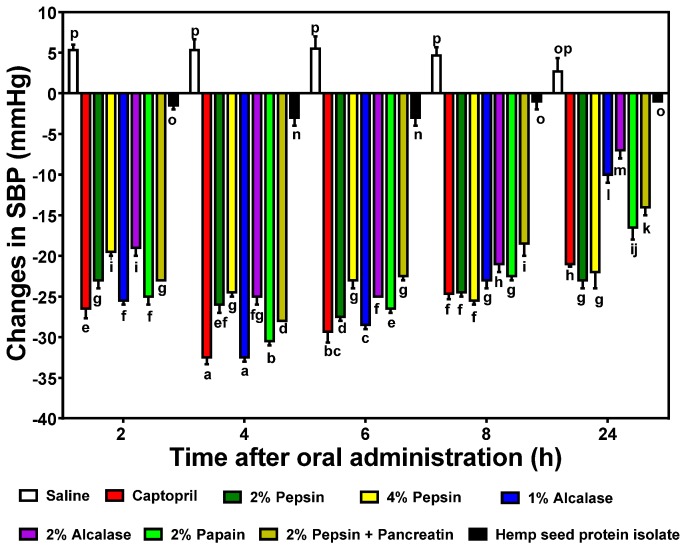
Time-dependent changes in systolic blood pressure (SBP) of spontaneously hypertensive rats (SHRs) after oral administration of different enzymatic hemp protein hydrolysates (HPHs) and hemp protein isolate (HPI).

Single oral administration (200 mg/kg bw) of the HPHs to SHRs resulted in varying but significant (*p* < 0.05) changes in SBP up to the 24 h period when compared to the negative control (Figure 6). The 1% alcalase was the most active with −32.5 ± 0.7 mmHg after 4 h, a result that is similar to that of the lower dose (10 mg/kg bw) drug (captopril). The 2% pepsin had significantly (*p* < 0.05) lower reductions after 2–6 h than the 4% pepsin, which is similar to the trend for the alcalase HPHs. However, both the 2% and 4% pepsin HPHs produced the longest-lasting effects with −23.0 ± 1.4 mmHg after 24 h of oral administration. The 1% and 2% alcalase HPHs had the least persistent effects with 24 h values of −10.0 ± 1.4 mmHg and −7.0 ± 1.4 mmHg, respectively. Interestingly, the SBP-reducing effects did not have a strict, direct relationship with observed *in vitro* ACE and renin inhibitions. However, the 2% papain with the highest ACE-inhibitory activity still produced highly significant (*p* < 0.05) SBP reductions, especially after 4 h (−30.5 ± 0.7 mmHg). However, the 2% alcalase which had low ACE and renin inhibition IC_50_ values also produced less SBP reductions when compared to other hydrolysates with higher *in vitro* inhibitory values.

## 4. Discussion

Apart from elucidating the arrangement of amino acids on a peptide chain, knowledge of the levels of amino acids present may also provide information on the structural basis for observed hydrolysate inhibitory activities. Only the cysteine level tended to differ among the hydrolysates, but the reason for the low cysteine level in 2% pepsin HPH is unclear. However, we can infer that at the 2% pepsin concentration, native protein structural restrictions may have limited proteolysis in areas that contained rigid disulfide bonds. At a higher 4% pepsin concentration, this restriction was probably overcome with greater availability of enzyme molecules, hence the cysteine content increased to a level comparable with other samples. The significantly higher DH value for the PP HPH may be attributed to the endo- and exo-peptidase activities of pancreatin, which enhances protein digestion through hydrolysis of more peptide bonds when compared to an enzyme with only endopeptidase activity. Moreover, predigestion with pepsin could have enhanced subsequent digestion by pancreatin and may have contributed to the high DH. Gonzalez-Garcia *et al.* [35] have also shown that Flavourzyme, an enzyme with similar endo- and exo-peptidase activities to those of pancreatin, also produced a hydrolysate with the highest DH. In contrast, the 2% pepsin digest had the lowest DH value by a significant margin at 4.40 ± 0.22%, which may be due to the specificity of the enzyme for mostly peptide bonds formed by aromatic amino acids. Alcalase and papain are non-specific endopeptidases, hence the high DH values when compared to pepsin. DH was higher when enzyme concentration increased, which is consistent with availability of more enzyme molecules for protein digestion.

The peptide sizes were estimated by SEC and the results showed distribution into four major peaks with approx. MW ranging from 300 to 9560 Da. These peptide peaks were most noticeable for the 2% pepsin HPH; increasing the enzyme concentration to 4% led to reduced intensities of the bigger peptide peaks (2775 and 9560 Da). The results support a higher proteolysis level at 4% pepsin, which is consistent with the higher DH when compared to the 2% pepsin. The alcalase and PP HPHs contained a minor peak with size >9560 Da (8–9 mL elution volume), which indicates a polypeptide that is probably resistant to digestion by these enzymes. The presence of minor peaks with size <305 Da (most likely free amino acids) were most intense in the PP, which could have been due to the presence of exoproteases in pancreatin. Overall, the data show that the HPI was highly susceptible to proteolysis and most of the liberated peptides were <9650 Da in size. The interrelationship between the MWs of hydrolysates and their bioactivities in human health application is very crucial in functional foods and nutraceutical formulations. Thus the presence of such low molecular weight peptides enhances the peptides’ absorption potential and ability to have bioactive effects during *in vivo* tests.

Variations in fluorescence intensity (FI) reflect structural conformation of proteins and peptides. Tyrosine and tryptophan are the main chromophores with emission maxima at 303 and 350 nm, respectively [42]. However, fluorescence intensity decreases and wavelength of maximum emission increases (red shift) upon exposure to aqueous environment because of water-dependent chromophore quenching. In contrast, fluorescence intensity increases and wavelength of maximum emission decreases (blue shift) when the chromophores are moved into a hydrophobic environment. Figure 3 shows two main peaks at 317 nm and 378 nm, which represent tyrosine and phenylalanine, respectively, in a hydrophilic environment. There was also a tryptophan shoulder peak at 338 nm, which suggests some tryptophan molecules in a more hydrophobic environment. Thus the tryptophan residues were in two different microenvironments, probably consisting of folded (338 nm) and open linear (378 nm) conformations. The HPI FI was very low, which indicates a denatured protein structure with most of the chromophores exposed to the hydrophilic environment. The higher FI of the peptides indicates close proximity between the tryptophan molecules and between the tyrosine molecules, which indicates some level of peptide–peptide interaction. These interactions could have been facilitated by the shorter and more flexible peptide conformations. The FI data confirms the structural differences between peptides, which have short-chain amino acid sequences, and the proteins with long amino acid sequences.

The better ACE-inhibitory activity of the 2% papain HPH may be due to the slightly higher proline content when compared to that of the other HPHs. This is because proline has been suggested as a potency-enhancing factor for ACE-inhibitory peptides based on the fact that the first two ACE-inhibitory peptides (VPP and IPP) isolated from fermented milk both contained proline [43]. However, proline content alone was not directly related to ACE-inhibitory activity of all the HPHs, which suggests that other peptide structural factors (e.g., proline position, presence of aromatic amino acids, or absence of disulfide bonds) may be responsible for the observed results. For example, the 2% pepsin HPH has very low cysteine level while the 2% PP HPH had slightly higher tyrosine level, both of which may have contributed to the poor ACE-inhibitory activities. The ACE-inhibitory levels are lower than some of the previously reported values for enzymatic food protein hydrolysates such as *Parkia speciosa* seed protein hydrolysates [24]. These differences may be due to the use of different protein materials for enzyme hydrolysis, since variations in the primary structure will lead to the liberation of peptides with distinct amino acid composition and sequences.

Unlike ACE, only very few reports [10,11,14,16,38,44] are available for peptide-dependent renin inhibition. A higher renin-inhibition seems to be related to phenylalanine content, since this amino acid was highest in the 2% pepsin, 4% pepsin and 2% PP HPHs. However, other structural features (e.g., amino acid arrangement and type) are likely to be involved in determining renin-inhibitory properties of the HPHs. A previous report has suggested the importance of branched-chain amino acids and aromatic amino acids in potentiating renin-inhibitory properties of food protein-derived peptides [45]. Generally, renin inhibitory activity of the hydrolysates was higher than ACE-inhibitory activity, which is different from other reports that showed a reverse trend [9,12,22,46]. Overall, these results confirm previously published literature data that indicate peptide-dependent ACE-inhibitory potency are more likely to be different from that renin inhibition. Since catalytic mechanisms differ between ACE and renin, molecules that have a strong inhibition against one enzyme may not necessary have the same inhibition level against the other enzyme.

Single oral administration of the HPHs to SHRs resulted in varying but significant (*p* < 0.05) changes in SBP up to the 24 h period when compared to the negative control (Figure 6). The 1% alcalase was the most active with −32.5 ± 0.7 mmHg after 4 h, a result that is similar to that of the lower dose drug (captopril). The 2% pepsin had significantly (*p* < 0.05) lower reductions than the 4% pepsin after 2–6 h, which is similar to the trend for the alcalase HPHs and indicates higher enzyme concentrations may not necessarily produce more effective antihypertensive peptides. The results are similar to the work of Alashi *et al.* [22] who also showed that alcalase and pepsin canola protein digests produced the most SBP reductions in SHR also at a 200 mg/kg bw dose. He *et al.* [9] also showed that an alcalase digest of rapeseed was the most effective SBP-reducing agent among several enzymes used. The results suggest that the lower renin-inhibition potency of the 2% papain HPH may have been compensated for by the higher ACE-inhibitory activity, hence strong reductions in SBP. The lack of direct correlation between *in vitro* and *in vivo* activities of the HPHs is consistent with previous reports [9,22]. The maximum SBP reductions obtained in this work are similar to the maximum value obtained for a sweet potato protein hydrolysate, which was administered at a higher 500 mg/kg bw dose [36]. Pistachio [17] and almond [19] protein hydrolysates at 1000 and 800 mg/kg bw doses, respectively, also produced lower SBP-reducing effects than the HPHs used in this work. In contrast to the HPHs, the unhydrolyzed HPI was less active (SBP values of −1 to −3 mmHg), which suggests that enzymatic predigestion converted the inactive proteins into bioactive peptides. Even though predigestion with pepsin alone or in combination with pancreatin produced SBP-reducing HPHs, the lack of effect after ingestion of HPI suggests that other *in vivo* factors (buffering capacity and duration of hydrolysis) may have prevented effective digestion and liberation of antihypertensive peptides in the SHR gastrointestinal tract. Similar works with pea [23] and rapeseed [9] have also shown better and faster SBP-reducing effects of predigested proteins when compared to undigested proteins.

## 5. Conclusions

The present study confirmed the dual *in vitro* ACE and renin inhibition capacity of enzymatic HSP digests. There was no relationship between the DH and ACE inhibitory activity of the protein hydrolysates, though renin inhibition tended to be inversely related to DH, which suggests longer hemp seed peptide chains may be more potent *in vitro* renin inhibitors than shorter chains. The DH and *in vitro* ACE or renin inhibition had no relationship with actual SBP-reducing effects in SHR, which suggests peptide amino acid sequence or synergistic effects between different peptides may be the determining factors for the observed antihypertensive potency. The significantly (*p* < 0.05) longer-lasting SBP-reducing effects of the pepsin digests, which also had the least DH (longer peptide chains) is an indication of reduced rate of absorption coupled with higher resistance to enzymatic clearance after absorption from the gastrointestinal tract. The alcalase digest had the fastest and highest SBP-reduction and may be combined with the papain digest (also highly potent with long-lasting effects) to produce a consistent SBP-lowering effect on a 24 h basis. However, a combination of pepsin and alcalase digests may also produce desirable 24 h SBP-reducing effects. Further studies are needed to determine the amino acid sequence of the most active HPH peptides (pepsin, alcalase, and papain) in order to perform structure-function studies. Overall, the results provide critical information on HPHs that may be used as active ingredients to formulate antihypertensive functional foods and nutraceuticals.
